# Directly Matching an MMIC Amplifier Integrated with MIMO Antenna through DNNs for Future Networks

**DOI:** 10.3390/s22187068

**Published:** 2022-09-19

**Authors:** Lida Kouhalvandi

**Affiliations:** Department of Electrical and Electronics Engineering, Dogus University, Istanbul 34775, Turkey; lida.kouhalvandi@ieee.org

**Keywords:** deep neural network (DNN), linearity, long short-term memory (LSTM), multiple-input and multiple-output (MIMO) antennas, multivariate newton’s method, monolithic microwave integrated circuit (MMIC), optimization

## Abstract

Due to the exponential growth of data communications, linearity specification is deteriorating and, in high frequency systems, impedance transformation leading to power delivering from power amplifiers (PAs) to antennas is becoming an increasingly important concept. Intelligent-based optimization methods can be a suitable solution for enhancing this characteristic in the transceiver systems. Herein, to tackle the problems of linearity and impedance transformations, deep neural network (DNN)-based optimizations are employed. In the first phase, the antenna is modeled through the DNN with using the long short-term memory (LSTM) leading to forecast the load impedances in the a wide frequency band. Afterwards, the PA is modeled and optimized through another LSTM-based DNN using Multivariate Newton’s Method where the optimal drain impedances are predicted from the first DNN (i.e., modeled antenna). The whole optimization methodology is executed automatically leading to enhance linearity specification of the whole system. For proving the novelty of the proposed method, monolithic microwave integrated circuit (MMIC) along with the multiple-input multiple-output (MIMO) antenna is designed, modeled, and optimized concurrently in the frequency band from 7.49 GHz to 12.44 GHz. The proposed method leads to enhancing the linearity of the transceiver in an effective way where DNN-based PA model gives rise to a solution for achieving the most optimal drain impedance through the modeled DNN-based antenna.

## 1. Introduction

Future wireless communication systems, including the fifth and sixth generation (5G and 6G) technologies, are growing day-by-day, resulting in exponentially high traffic data [1,2,3,4,5,6]. To provide a reliable communication system, the overall performances of power amplifiers (PAs) and antenna as active and passive components play important roles [7,8,9,10]. Typically, PAs exist near the antennas to transmit a high-power signal, and are challenging in terms of various specifications, especially linearity [11]. This is a big challenge in radio frequency (RF) designs, and designers must find a well-suited solution for optimizing the overall communication systems to balance the linearity trade-off.

The main structure of communication systems consists of concurrent existence of passive and active devices. Hence, due to the various unbalanced effects generated from these devices, additional efforts with strong optimization methods are needed to improve the overall performance of systems [12,13,14,15]. Recently, various papers have studied cointegrated antenna with amplifiers leading to an enhancement of the overall performances, mainly in terms of energy efficiency and bandwidth. In [16], a methodology is explained for designing cointegrated antenna with an amplifier through balanced multiport feeding. The presented feeding with the optimization method improved the bandwidth of the overall system. In another study [17], a PA-aware precoding approach is suggested for investigating the compensation of the PA along with the multiple-input and multiple-output (MIMO) antenna to guarantee transmission quality. To enhance the energy efficiency of arrays under PA designs, an approach is offered in [18] where it the transmit duration with powers is determined. A nonlinear optimization, based on the far-field is proposed in [19] where the radiating part is optimized through an artificial neural network (ANN). In [20], the target is to find the optimal matching networks for enhancing the power transfer efficiency among arbitrary impedances to achieve the optimal transmission. In the domain of radar, a joint synthesis is presented in [21] for ensuring the compatibility with amplifiers. In another study ([22]), a solution for resource allocation is described that includes antenna array and PAs at the user and relay nodes by proposing the low-complexity suboptimal relay selection. In [23], the nonlinearity of PA is modeled through the Saleh model to be used in the satellite-terrestrial network leading to provide optimal beamforming. The suboptimal algorithm is employed in [24], for optimal MIMO precoding by considering the consumed power from the PA side. Herein, finding an optimal drain impedance matched to the antenna with high performance specification, especially in terms of linearity, requires intelligent-based optimization methods [25]. Recently, machine learning paves the way for signal processing, RF, and very-large-scale integration (VLSI) designs as it is able to model and optimize complex configurations by providing the relationship between input and output specification [26].

In communication systems, due to the simultaneous existence of PAs and antennas, it is not straightforward to achieve the optimal load impedance from antenna side that is connected to the transistor’s drain impedance. In the previously reported studies, this problem with the linearity investigation is not considered. Hence, to tackle these drawbacks, we propose modeling of the antenna and PA through deep neural networks (DNNs) for finding the optimal load impedance, and optimizing the linearity specification of whole system based on the ’Multivariate Newton’s’ Method [27]. To the best of the author’s knowledge, a joint methodology for modeling and optimizing cointegrated antenna and PA through DNNs is proposed for the very first time in the literature. In the first phase, the antenna is modeled through the long short-term memory (LSTM)-based DNN, leading to a prediction of the load impedance, including real and imaginary parts, in a large bandwidth. Afterwards, the PA is modeled through LSTM-based DNN for optimizing the linearity performance of the system where it is optimized through the predicted optimal load impedances from the modeled antenna. For the proposed methodology, two electronic design automation (EDA) tools with a numerical analyzer (as MATLAB) are used where Microwave Studio (Dassault Systèmes) and ADS are executed for designing antenna and PA, respectively. For sizing the existed components, the genetic algorithm (GA) is employed, and for providing the optimal hyperparameters, bayesian optimization (BO) is used. The contributions of this manuscript can be summarized as follows:

- Modeling the antenna with LSTM-based DNN leading to estimate the real and imaginary parts of load impedances over the large bandwidth;
- Directly matching the antenna into the drain’s optimal impedance;
- Modeling the PA with the LSTM-based DNN through multivariate Newton’s method;
- Linearity improvement of the overall system by cointegration of antenna and amplifier as passive and active devices, respectively.

This paper is organized as follows: Section 2 presents the proposed methodology for modeling antennas and amplifier, leading to enhanced linearity specification in the communication systems. Section 3 describes the practical implementation of the proposed method. Section 4 is devoted to providing the simulation results of optimized and modeled antenna with amplifier. Finally, Section 5 concludes this work.

## 2. Proposed Optimization Method

**Figure 1:**
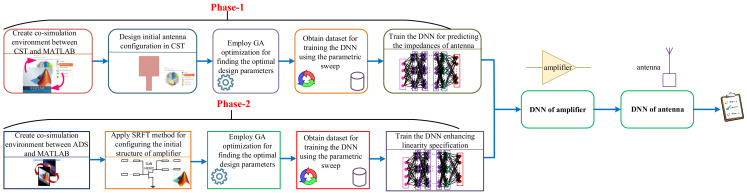
Comprehensive overview around the proposed methodology where two LSTM-based DNNs are employed for modeling antenna and PA.

This section presents the optimization-oriented algorithm consisting of two sequential phases for: (i) modeling the antenna with the LSTM- based DNN, and (ii) modeling and optimizing the PA design in terms of linearity where it is in relation with the modeled antenna and it is optimizing with respect to the predicted load impedances from the antenna side. The DNNs are the networks that enable more accurate prediction of the targeted data than shallow neural networks (SNNs) [28], and LSTM-based DNN is employed for predicting the multi-segment frequency-series data in the determined bandwidth.Training DNNs with more than 90% testing accuracy requires two important challenging factors: one is to have a suitable amount of dataset, and the other one is to achieve optimal hyperparameters [29,30]. This section provides detailed explanations about the proposed methodology, leading us to enhance the linearity specification in the communication systems. In the trained two DNNs (in phase 1 and phase 2), the used activation function and loss function are the rectified linear unit (ReLU) and mean squared error, respectively. Algorithm 1 (at the end of this section) describes the steps of applied method, and Figure 1 presents the general overview around the two phases of intelligent-based optimization method through DNNs.

### 2.1. LSTM-Based DNN for Modeling Antenna (Phase-1)

**Figure 2:**
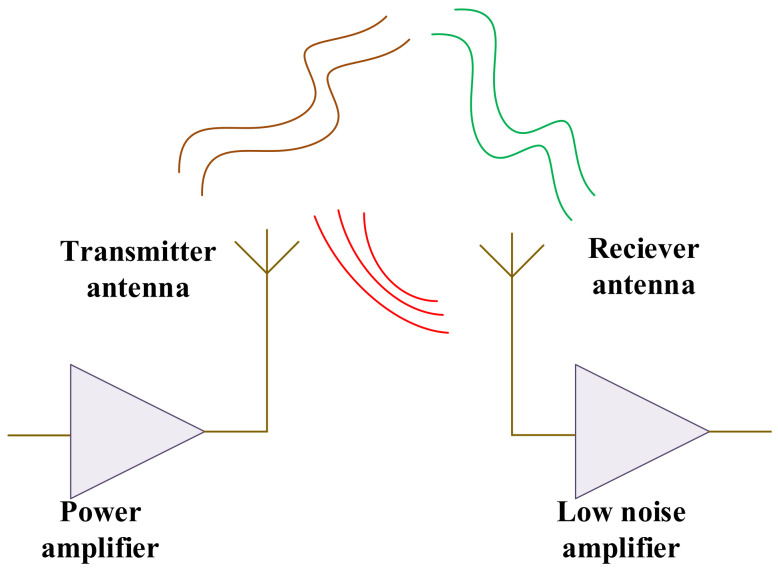
A typical structure of any communication system includes PAs and antennas.

Antennas play important role in both transmitting and receiving signals in communication systems. They can either radiate or receive electromagnetic waves as the essential data. Hence, strong and high-performance antennas are required, essentially enabling us to convert electrical energy into electromagnetic waves and vice versa. Figure 2 presents the PA and antenna as active, and passive devices are the essential blocks in the communication systems.

**Figure 3:**
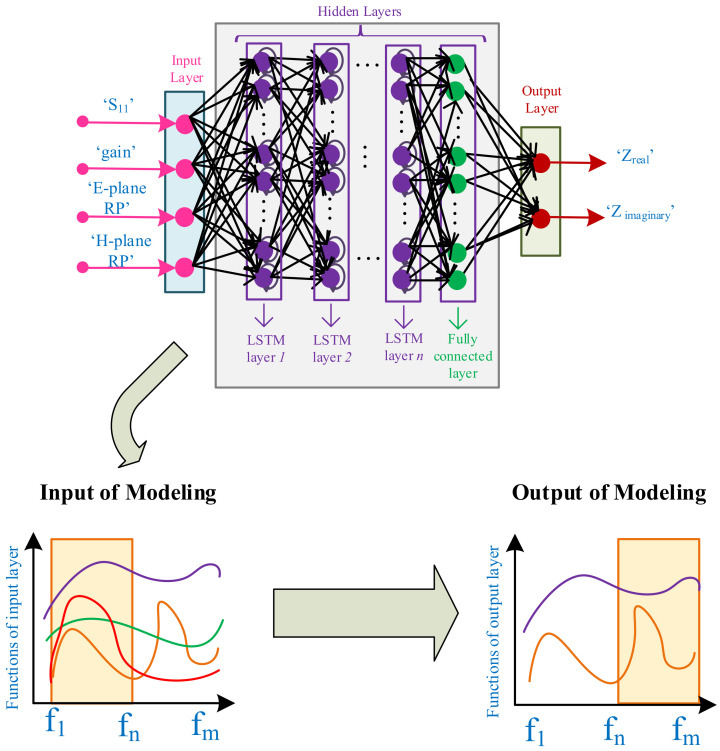
LSTM-based DNN for modeling the antenna and predicting the extended impedances in the frequency domain.

Matching the antenna to the drain’s optimal impedance such that the performance specifications are high is challenging. Hence, a strong method is needed for modeling the antenna to facilitate the overall performance of the amplifier. Intelligent-based methods that include DNNs are the most promising methods for modeling RF designs [31,32]. Therein, modeling the antennas, especially with complex structures, can be executed through DNNs. From another perspective, the antenna coupling can influence the integrated amplifiers, and some antenna elements can recognize negative impedances [33]. In this case, a well-designed amplifier that is matched with optimal drain impedances through LSTM-based DNNs can be a good solution. Figure 3 describes the antenna modeling approach for predicting the load impedances, including real and imaginary parts, over the large frequency band. In this paper, as the first phase, we present antenna modeling procedure through LSTM-based DNN.

As all the proposed methodology is performed automatically and based on the DNN constructions, a combination between EDA tools and numerical analyzer is required. Hence, for modeling the antenna, an automated environment between Microwave Studio (Dassault Systèmes) and MATLAB are created (Step-1). Afterwards, the initial structure of antenna is developed by means of the CST tool (Step-2). Determining the design parameters after constructing the initial configuration of antenna is a major concern allowing us to influence the output specification of the antenna. In this case, we employ the GA method that is based on the natural selection, and continuously alters the population of individual possibilities [34,35,36] (Step-3). After constructing the configuration of the antenna and finding the optimal design parameters, it is time to train the LSTM-based regression DNN, aiming to predict the extended load impedances of antenna in the interested frequency band. The main requirement of DNN construction is to provide a suitable sized dataset that includes training and testing data. For this case, a parametric sweep (i.e., random iteration) is employed for altering the design parameters and achieving the outcomes as $S_{11}$, gain, radiation patterns in E-plane and H-plane and load impedances (i.e., real and imaginary parts) for half of the full targeted bandwidth (Step-4). Another significant process in DNN construction is finding the hyperparameters. To achieve the optimal hyperparameters, we employ the BO method that enables us to achieve the optimal number of hidden layers with number of neurons in each layer. The BO is applied due to its capability in doing optimization over the continuous domains [37,38,39,40] (Step-5). As Figure 3 shows, each neural network structure consists of three sections, namely as input layer, hidden layers, and output layer. In our proposed DNN, the input layer defines important characteristics of antenna as $S_{11}$, gain, and radiation patterns. The output layer predicts the real and imaginary parts of the impedances generated from the antenna over the large bandwidth. Equation (1) presents the definition of training DNN where it requires data such as training input data ($X_{\mathrm{Train}}$) and training output data ($Y_{\mathrm{Train}}$). Afterwards, testing data ($X_{\mathrm{Test}}$) is needed for determining the accuracy of trained DNN (Equation (2)). In modeling DNN, the output specifications of antenna are generated from the CST side and MATLAB manages all the provided data, enabling us to manage the optimization process. (Step-6).

(1) $$\mathrm{net}=\mathrm{trainNetwork}(X_{\mathrm{Train}},Y_{\mathrm{Train}},\mathrm{layers})$$

(2) $$Y_{\mathrm{Pred}}=\mathrm{predict}\left(\mathrm{net},X_{\mathrm{Test}}\right)$$

### 2.2. Linearity Optimization through DNN Conjoined to the Modeled Antenna (Phase-2)

**Figure 4:**
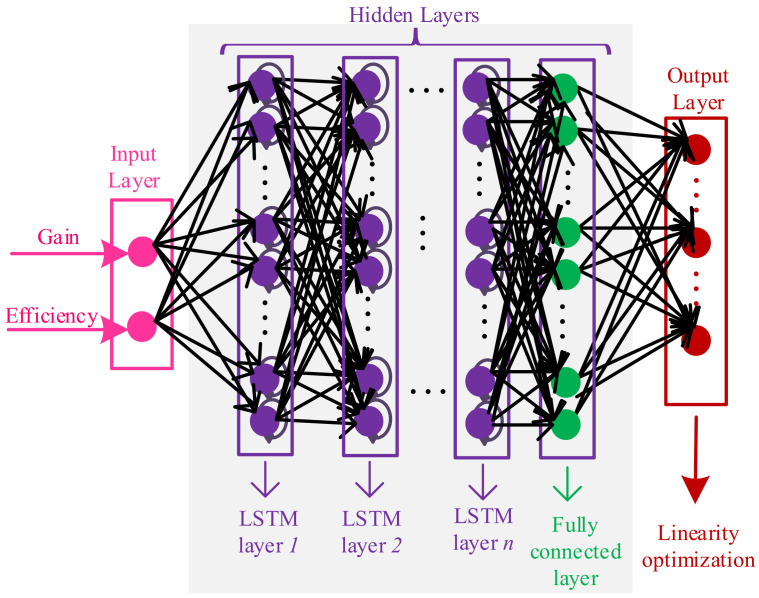
PA modeling through DNN for optimizing linearity connected to the modeled antenna.

The PAs are other key components in the manipulation of the RF power signals in base stations, especially in 5G systems [41,42]. By improving the technology type, for example from 5G to 6G technology, linearity specification is becoming more critical. Hence, advanced modeling and optimization methods are required for enhancing the overall performance. Figure 4 presents an overview of the proposed method for optimizing the linearity specification of amplifier connected to the modeled antenna.

After modeling the antenna through the DNN, it is time to model and optimize the PA in terms of linearity. For this case, a co-simulation environment between ADS and MATLAB is created (Step-7). Then, the initial structure of the PA with component values are achieved through the simplified real frequency technique (SRFT) [43]. From the SRFT method, the topology of PA, including input and output matching networks, with initial values are obtained. Afterwards, the GA method is applied for improving the output performance of PA (Step-8). As described in Section 2.1, the dataset must be provided for training the DNN. Herein, the parametric sweep is performed by altering the component values randomly and the output specifications of PA in terms of power added efficiency (PAE(%)), gain ($G_{p}\left(\mathrm{dB}\right)$), and intermodulation (IMDs (dBc)) as (third IMD (${\mathrm{IMD}}_{3}$), fifth IMD (${\mathrm{IMD}}_{5}$), and seventh IMD (${\mathrm{IMD}}_{7}$)) are extracted automatically (Step-9).

Optimizing the function for linearity specification is somewhat complex and it requires additional efforts because ADS generates data for each frequency point. Hence, to facilitate the IMD computations, the average mean values (M) for the high and low sides of IMD levels are calculated for the third, fifth, and seventh orders as presented in (3)–(5). Afterwards, we propose using the ’Multivariate Newton’s Method’ [27] for providing the approximate function (i.e., representation) of linearity specification.

(3) $${\mathrm{IMD}}_{3}=\left[\left({\mathrm{IMD}}_{3(\mathrm{low})}\right)+\left({\mathrm{IMD}}_{3(\mathrm{high})}\right)\right]/2$$

(4) $${\mathrm{IMD}}_{5}=\left[\left({\mathrm{IMD}}_{5(\mathrm{low})}\right)+\left({\mathrm{IMD}}_{5(\mathrm{high})}\right)\right]/2$$

(5) $${\mathrm{IMD}}_{7}=\left[\left({\mathrm{IMD}}_{7(\mathrm{low})}\right)+\left({\mathrm{IMD}}_{7(\mathrm{high})}\right)\right]/2$$

Newton’s method is optimizing $F(x_{1},x_{2},\cdots ,x_{n})$ in n variables, $x=(x_{1},x_{2},\cdots ,x_{n})$ to find the optimal points in the domain of F with determined initial guess values. This method firstly extracts the derivative of F(x) by using Jacobian matrix as presented in (6).

(6) $$\partial F=\left[\begin{array}{ccc} \frac{\partial F_{1}}{\partial x_{1}} & \cdots & \frac{\partial F_{1}}{\partial x_{n}}\\ \cdots & \cdots & \cdots \\ \frac{\partial F_{m}}{\partial x_{1}} & \cdots & \frac{\partial F_{m}}{\partial x_{n}} \end{array}\right]$$

By doing IMD simulation in ADS for each of the frequency points, various IMDs can appear. Table 1 describes the 3rd, 5th, and 7th order IMD levels in dBc (i.e., ${\mathrm{IMD}}_{3}$, ${\mathrm{IMD}}_{5}$, and ${\mathrm{IMD}}_{7}$) for each frequency point. Newton’s method is employed to our problem starting from Equations (3)–(5). Herein, initial IMD values with suitable function representation for each IMD must be prepared. Firstly, the initial values are achieved using the *Standard Deviations* for values in each column of ${\mathrm{IMD}}_{3}$, ${\mathrm{IMD}}_{5}$, and ${\mathrm{IMD}}_{7}$. To obtain a clear idea on how to construct the function, Equation (7) presents the immediate instance of constructing ${\mathrm{IMD}}_{3}$ representation. For the presented IMD value at each frequency point, (i.e., $a_{1}+a_{2}+\cdots +a_{n}$), and average mean (M) with variance ($\sigma^{2}$) values are calculated, and then the square root of Variance as Standard Deviation (σ) is obtained.

(7) $$\begin{array}{c} M_{\mathrm{IMD}3}=(a_{1}+a_{2}+\cdots +a_{n})/n\\ \sigma_{\mathrm{IMD}3}^{2}=(a_{1}^{2}+a_{2}^{2}+\cdots +a_{n}^{2})/n\\ \sigma_{\mathrm{IMD}3}=\sqrt{\sigma_{\mathrm{IMD}3}^{2}} \end{array}$$

Secondly, a function representing each IMD behavior must be constructed by determining the suitable coefficient at each frequency point. For this purpose, *fit* function in MATLAB [44] is applied to the data points of each frequency presented in Table 1 and three functions representing third, fifth, and seventh IMDs are constructed. For example, third order IMD can be represented by: $f_{\mathrm{IMD}3}={\mathrm{Aa}}_{1}+{\mathrm{Ba}}_{2}+\cdots +{\mathrm{Na}}_{n}$ where A, B,…, and  N are the coefficients achieved from ’fit’ function.

After achieving initial values for IMDs (i.e., $\sigma_{\mathrm{IMD}3}$, $\sigma_{\mathrm{IMD}5}$, and $\sigma_{\mathrm{IMD}7}$ ) and constructing IMD equations such as $f_{\mathrm{IMD}3}$, $f_{\mathrm{IMD}5}$, and $f_{\mathrm{IMD}7}$, Newton’s Method [45] is applied (here implemented in MATLAB). This method results in computing three optimized critical points as: ${\mathrm{IMD}}_{3\mathrm{opt}}$,${\mathrm{IMD}}_{5\mathrm{opt}}$, and ${\mathrm{IMD}}_{7\mathrm{opt}}$. By using these data and applying [44] curve, an accurate optimized function (8) representing linearity is constructed where $L_{1}$, $L_{2}$, and $L_{3}$ are the constant values (Step-10).

(8) $$\mathrm{Linearity}(\mathrm{dBc})=L_{1}\left({\mathrm{IMD}}_{3\mathrm{opt}}\right)+L_{2}\left({\mathrm{IMD}}_{5\mathrm{opt}}\right)+L_{3}\left({\mathrm{IMD}}_{7\mathrm{opt}}\right)$$

By the generated dataset and the constructed target function (i.e., linearity), the LSTM-based DNN is trained as the procedure presented in Step-6 and the BO method is applied for finding the optimal hyperparameters (Step-11). Finally, with two LSTM-based DNNs such that one is used for modeling PA and the other is for the modeling antenna, the whole system is optimized. The modeled PA is optimizing the linearity performance of whole system by the directly matching to the optimal predicted impedance from the modeled antenna through DNN (Step-12).
**Algorithm 1** Proposed methodology for concurrently modeling antenna and amplifiers in the communication systems leads to enhanced linearity specification.**Phase 1 (Antenna modeling)** ** 1**: Create co-simulation environment between CST and MATLAB; ** 2**: Design the initial structure of the antenna; ** 3**: Employ the GA method for optimizing the design parameters; ** 4**: Achieve a suitable dataset size, i.e., training and testing dataset, by performing the parametric sweep; ** 5**: Achieve the optimal hyperparameters of DNN used for modeling the antenna through BO method; ** 6**: Train the LSTM-based DNN enabling the prediction of the optimal imepdance that is perfectly matched to the drain impedance transistor over the large frequency band; ** Phase 2 (PA modeling and optimization in terms of linearity along with the modeled antenna)** ** 7**: Construct co-simulation environment between ADS and MATLAB; ** 8**: Configure the PA structure with the SRFT method and run the GA method to obtain the optimal design parameters; ** 9**: Obtain the dataset in terms of gain, efficiency, and IMDs for constructing the LSTM-based DNN; ** 10**: Employ Multivariate Newton’s Method for optimizing the linearity specification; ** 11**: Train the regression DNN for the PA where the optimal hyperparameters are achieved using the BO method; ** 12**: Run the modeled antenna and PA concurrently for optimizing the whole linearity specification of communication systems over the determined frequency band.


## 3. Practical Execution for Training Two DNNs

Employing a successful methodology requires a suitable implementation environment. For this case, we start the proposed methodology at CPU environment with the characteristics of Intel Core i7-4790 CPU @ 3.60 GHz with 32.0 GB RAM. In this study, we design and optimize MIMO antenna with a monolithic microwave integrated circuit (MMIC) design that is a class-AB amplifier.

**Figure 5:**
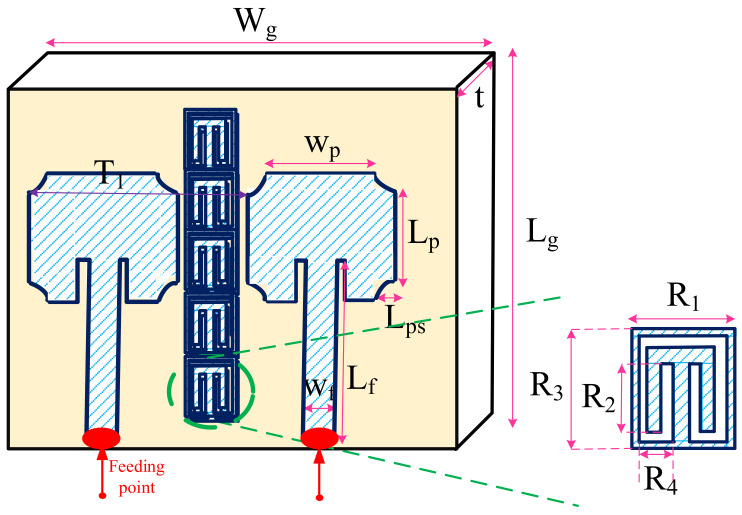
MIMO antenna structure.

In the first phase, the structure of MIMO is constructed, and after employing the GA method the optimal design parameters are obtained. Figure 5 presents the MIMO antenna structure with the ground plane of 120 mm and 120 mm, along x and y directions, respectively and Table 2 presents the optimal values of parameters. The presented MIMO antenna consists of two single antennas with rings between two patch-like radiators aiming to reduce the surface waves coupling between them. The MIMO antenna is implemented on the FR-4 substrate with $\varepsilon_{r}$ of 4.3 and and loss tangen of δ = 1 × 10 ${}^{-3}$.

After sizing the antenna, the parametric sweep is employed with the different ranges of ∓5%, and ∓10% to obtain the suitable amount of dataset, includíng training and testing data. Afterwards, the hyperparameters of DNN are achieved through the BO method where 3 LSTM layers with 135 neurons are used for modeling the antenna with the use of DNN. In total, 1500 data are achieved at various frequencies, where the input layer includes characteristics are: $S_{11}$, gain, and E-plane with H-plane radiation patterns. The output layer also presents the real impedances ($Z_{\mathrm{real}}$) and imaginary impedances ($Z_{\mathrm{imaginary}}$) in each frequency. The training accuracy of the DNN is around 0.085.

**Figure 6:**
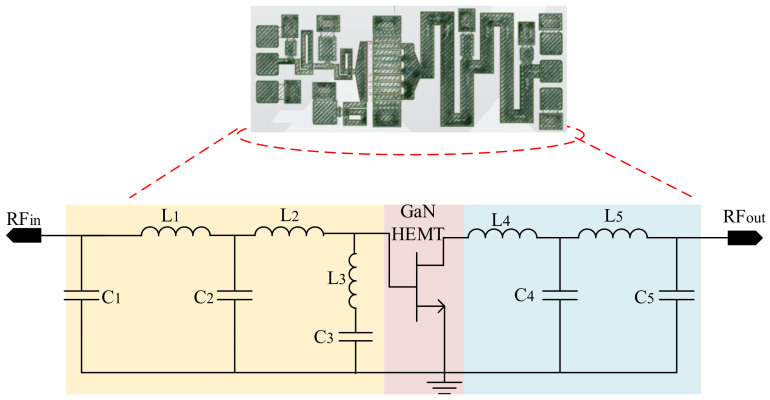
MMIC design and optimization through DNN.

At the second phase, the amplifier is modeled through DNN based on the predicted optimal impedances from modeled antenna that are directly matched to the drain impedance of transistor. For configuring the amplifier, the SRFT method is employed with the gate and drain impedances achieved from load-pull measurement. Figure 6 presents the configuration of MMIC, which is the class-AB amplifier, and the optimal parameters are achieved through the GA method. Table 3 presents the optimal values of the components included in the design of MMIC. After extracting the MMIC structure with the design parameters, the parametric sweep is performed within the range of ∓5%, and ∓10% (like the antenna modeling) and 2600 data in terms of gain, efficiency, and third, fifth, and seventh IMDs at various frequencies are extracted. Afterwards, the multivariate Newton’s method (at the output layer) is applied for optimizing linearity specification. The BO is employed to determine the optimal hyperparameters of the second DNN, resulting in 4 LSTM layers with 100 neurons in each layer. The training accuracy for the modeled amplifier is around 0.093.

## 4. Simulation Results of the Optimized System with a Combination of MMIC and MIMO Antenna Designs

This section is devoted to presenting the outcomes achieved from the modeled MIMO antenna and MMIC design enabling us to optimize overall linearity specification of the system.

**Figure 7:**
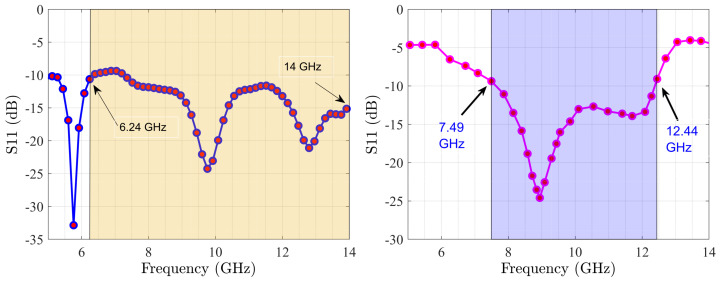
$S_{11}$ of optimized MIMO (**left**) with $S_{11}$ of optimized MMIC design (**right**) through DNNs.

**Figure 8:**
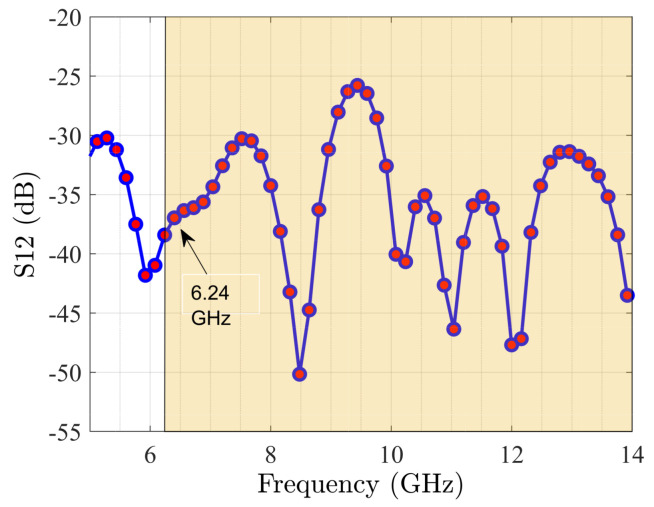
$S_{12}$ of optimized MIMO.

**Figure 9:**
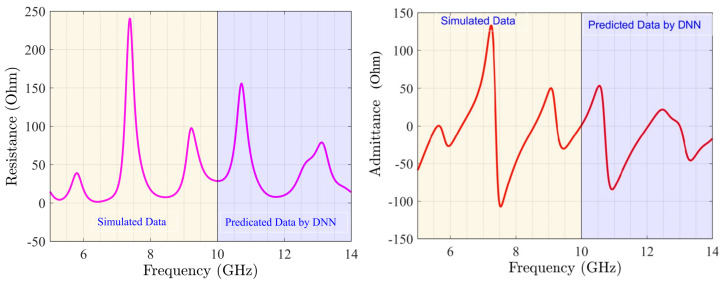
Predicated resistance (**right**) and reactance (**left**) of optimized MIMO through DNN.

**Figure 10:**
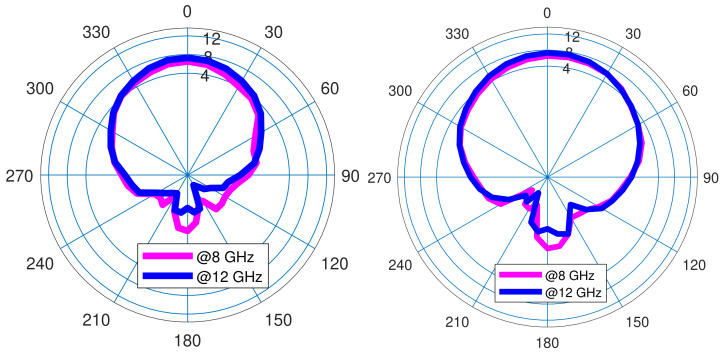
Radiation pattern of MIMO antenna at $f_{1}=8$ GHz (pink), $f_{2}=12$ GHz (blue); ϕ=0 (**left**), $\phi =90^{\circ }$ (**right**).

The main challenge in communication systems is to have amplifiers and antennas at the common bandwidth with the perfectly matched impedance. The presented MIMO covers the frequency band of 6.24 GHz to 14 GHz, as Figure 7 shows, and MMIC design enhances the output power at the frequency range of 7.49 GHz to 12.44 GHz. Herein, the common frequency bandwidth is 7.49 GHz to 12.44 GHz for the whole system. Another important specification of the antenna is the $S_{12}$ specification, as Figure 8 shows. Figure 9 describes the resistance ($Z_{\mathrm{real}}$) and reactance ($Z_{\mathrm{imaginary}}$) results achieved from the simulations from 5 GHz to 10 GHz and also the predicted outcomes through trained DNN from 10 GHz to 14 GHz. Figure 10 shows that the radiation patterns at different frequencies have been achieved according to the value of ϕ=90 and ϕ=0, respectively.

**Figure 11:**
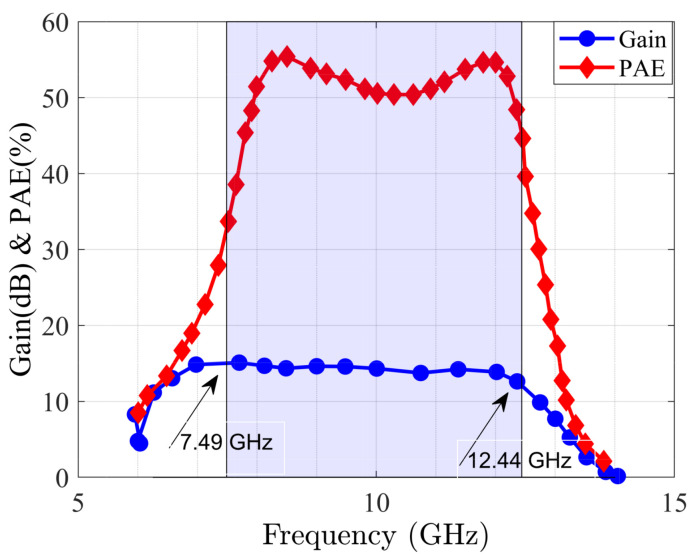
Gain and PAE of optimized MMIC design through DNN at 38 dB output power.

**Figure 12:**
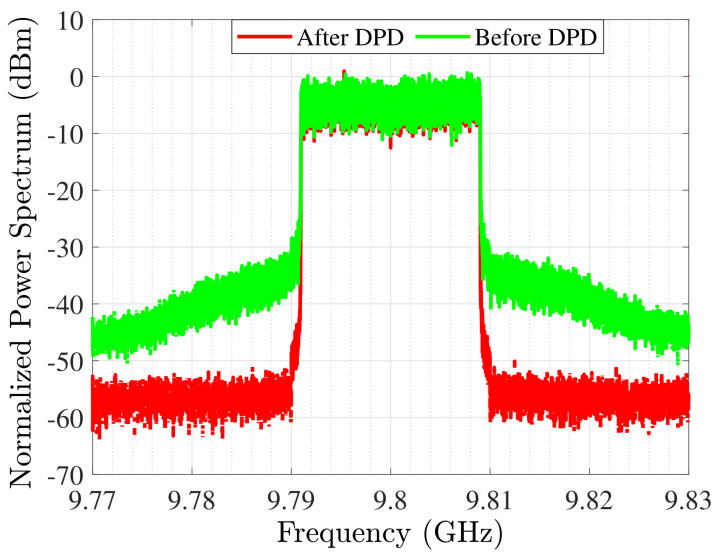
Output spectrum presentation with 20 MHz LTE signal before and after DPD.

**Figure 13:**
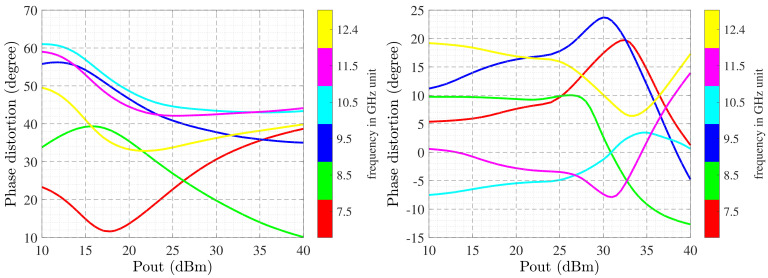
Linearity comparison before (**right**) and after doing optimization (**left**).

After modeling the MIMO antenna, the class-AB amplifier is deigned and optimized through the WIN 0.25 μm Gallium Nitride (GaN) process technology and is biased at 28 V and 100 mA/mm. The SRFT method causes inductor-capacitor (LC) ladders at the input and output matching networks where the optimized amplifier leads a linear gain of more than 10 dB with more than 50 % power added efficiency (PAE) at the determined bandwidth with 38 dB output power (see Figure 11). Figure 12 presents the modulated signal response of amplifier after optimization in two cases: before digital pre-distortion (DPD) and after DPD. After employing the proposed methodology, the normalized power spectrum can reach the acceptable level, as desired. The IMDs are occurred due to the amplifier non-linearities. Phase distortion is a suitable specification for considering this effect [46]. Herein, Figure 13 presents the phase distortion before and after modeling at various frequencies. As shown, before optimization the phase distortion is around 70${}^{\circ }$ (i.e., between $-10^{\circ }$ and 60${}^{\circ }$). However, after optimization the variation is around 30${}^{\circ }$, at the range of $-10^{\circ }$ and 20${}^{\circ }$. In this case, there is around 55% improvement from the linearity specification by the existence of amplifier and antenna as active and passive devices, respectively.

Table 4 compares the very recently published methods for designing and optimizing a hybrid design that includes both antenna and amplifier. Our proposed method is based on the DNNs that: (1) provide an automated optimization process, (2) apply a multi-objective optimization method, (3) predict the impedances of antenna over the extended bandwidth without additional efforts, (4) enhance the linearity specification of overall system by considering the generated impedances from antenna side, and (5) provide an optimization environment where active and passive designs can be designed and optimized conjointly. In the recently published literature, such contributions are lacking. In contrast, our proposed method leads to reduced computational complexity.

## 5. Conclusions

The linearity specification is a crucial aspect in the communication systems where optimization of this outcome is not straightforward and requires substantial efforts. For the very first time in the literature, we present a novel methodology for concurrently optimizing active and passive devices leading to enhanced overall performance. In this study, we design MIMO antenna with MMIC design as passive and active devices, respectively. Firstly, the antenna is modeled through DNN which forecasts the impedances that can be directly matched to the drain impedance of a used transistor at large bandwidth. Afterwards, the MMIC design, a class AB amplifier, is modeled through the DNN where it is optimized with respect to the predicted impedances through modeled antenna. In the second DNN, the multivariate newton’s method is employed, enabling us to optimize the linearity specification of overall system. To prove the novelty of the proposed method, MMIC design with MIMO antenna are concurrently designed and optimized in the frequency band of 7.49 GHz to 12.44 GHz. Our proposed methodology can be developed by: (1) considering various types of neural networks, (2) using more reliable transistor models in the MMIC designs, (3) altering the feeding points of the antenna, (4) considering the non-orthogonal multiple access (NOMA) scheme for 5G networks, (5) further investigating the effect of antenna coupling on the PAs.

## Figures and Tables

**Table 1:** Summarized IMD level data obtained from (3)–(5).

Frequency	${\mathrm{IMD}}_{3}$ (3)	${\mathrm{IMD}}_{5}$ (4)	${\mathrm{IMD}}_{7}$ (5)
$f_{1}$	$a_{1}$	$b_{1}$	$c_{1}$
$f_{2}$	$a_{2}$	$b_{2}$	$c_{2}$
…	…	…	…
$f_{n}$	$a_{n}$	$b_{n}$	$c_{n}$

**Table 2:** Design parameters of optimized MIMO antenna in Figure 5.

Design Parameters	Value (mm)	Design Parameters	Value (mm)
$T_{1}$	22.5	$W_{p}$	11
$L_{p}$	8.99	$L_{\mathrm{ps}}$	1.4
$L_{f}$	18.1	$W_{f}$	3.2
$R_{1}$	5.2	$R_{2}$	3.6
$R_{3}$	6.2	$R_{4}$	1.7
$W_{g}$	45	$L_{g}$	38
t	1.65		

**Table 3:** Design parameters of optimized MMIC, class-AB amplifier, in Figure 6; The unit of each inductor and capacitor are nH and pF, respectively.

Design Parameters	Value	Design Parameters	Value
$L_{1}$	0.46	$C_{1}$	0.58
$L_{2}$	0.29	$C_{2}$	1.51
$L_{3}$	0.64	$C_{3}$	2.72
$L_{4}$	0.71	$C_{4}$	1.2
$L_{5}$	0.68	$C_{5}$	0.63

**Table 4:** Summary of various methodologies employed for hybrid design of antennas with amplifiers in recent publications.

Ref.	Method	Goal(s) of Paper
[16]	Balancing multiport feeding of the cavity-backed patch antenna	- Providing the optimal loading conditions for doherty PA
[17]	PA-aware precoding method by convex optimization	- Exploiting the high-dimensional degrees of freedom;
		- Enhancing transmission quality
[18]	Coherent and non-coherent beamforming consideration	- Maximizing the energy efficiency
[19]	ANN-based optimization	- Optimizing the radiation and nonlinear performances
[20]	Nonlinear loadpull simulations	- Maximizing efficiency and bandwidth
[21]	Iterative procedure	- Optimizing radar performance in signal-dependent interference
[22]	Analytical expressions for the nonlinear distortion	- Enhancing resource allocation problem
[23]	Saleh PA-model for nonlinearity and the large scale satellite channel parameters	- Maximizing the achievable rate of the satellite system
[24]	Successive convex approximation	- Considering the capacity of the MIMO channel
[25]	Methodology based on the signal-to-noise ratio	- Optimizing energy Efficient
This work	Two LSTM-based DNNs with Multivariate Newton’s Method	- Modeling of antenna and amplifier through LSTM-based DNNs;
		- Predicting the extended impedance over the large bandwidth;
		- Achieving the optimal drain impedances;
		- Enhancing the linearity specification of the overall system.

## Data Availability

Not applicable.

## Abbreviations

The following table is provided in this manuscript for easy accessing to various definitions with their phrases:

ANN	Artificial neural network
BO	Bayesian optimization
DPD	Digital pre-distortion
DNN	Deep neural network
EDA	Electronic design automation
5G	Fifth generation
GA	Genetic algorithm
GaN	Gallium Nitride
IMD	Intermodulation
LC	Inductor-capacitor
LSTM	Long short-term memory
MMIC	Monolithic microwave integrated circuit
MIMO	Multiple-input multiple-output
M	Mean values
NOMA	Non-orthogonal multiple access
PA	Power amplifier
PAE	Power added efficiency
ReLU	Rectified linear unit
SRFT	Simplified real frequency technique
6G	Sixth generation
VLSI	Very-large-scale integration
RF	Radio frequency
$X_{\mathrm{Train}}$	Training input data
$Y_{\mathrm{Train}}$	Training output data
$X_{\mathrm{Test}}$	Testing data
$G_{p}$	Gain
$\sigma^{2}$	Variance
σ	Standard Deviation
$Z_{\mathrm{real}}$	Real impedances
$Z_{\mathrm{imaginary}}$	Imaginary impedance
